# PICALM rs3851179 Variants Modulate Left Postcentral Cortex Thickness, CSF Amyloid β42, and Phosphorylated Tau in the Elderly

**DOI:** 10.3390/brainsci12121681

**Published:** 2022-12-07

**Authors:** Zhiwei Wu, Yiwen Yang, Ziyang Song, Mengya Ma, Mengmeng Feng, Yuanqing Liu, Hanqi Xing, Yue Chang, Hui Dai

**Affiliations:** 1 Department of Radiology, First Affiliated Hospital of Soochow University, Suzhou 215006, China; 2 Institute of Medical Imaging, Soochow University, Suzhou 215006, China; 3 Suzhou Key Laboratory of Intelligent Medicine and Equipment, Suzhou 215123, China

**Keywords:** Alzheimer’s disease, PICALM, rs3851179, amyloid β, phosphorylated tau

## Abstract

PICALM rs3851179, one of the genes most frequently linked to susceptibility of late-onset Alzheimer’s disease (LOAD), plays a crucial role in regulating amyloid precursor protein, and amyloid β (Aβ) transcytosis. To explore the effects of PICALM and AD continuum stage on cortex thickness, CSF Aβ, and tau, 188 cognitively normal controls, 261 MCI patients, and 140 early LOAD patients were recruited, and each group was divided into rs3851179 A-carriers and GG-carriers. A full factorial ANCOVA was used to analyze the main effects and interactive effects of AD continuum stage, and PICALM. The interactive effects of AD continuum stage and PICALM on cortex thickness and CSF biomarkers were not significant. The main effect of PICALM was significant on the left postcentral cortex thickness, and the cortex thickness of A-carriers was less than that of GG-carriers. The rs3851179 A-carriers displayed higher Aβ42 levels and Aβ42/40 ratios, and lower P/T–tau ratios, compared with GG-carriers. A higher MMSE score was found in A-carriers among the LOAD patients. In conclusion, the main effects of PICALM were independent of AD continuum stage, and PICLAM rs3851179 genotypes may modulate left postcentral cortex thickness, Aβ42 level, and P/T–tau ratio. The rs3851179 A-allele may protect the cognitive function of LOAD patients.

## 1. Introduction

Alzheimer’s disease (AD) is the main cause of dementia, and is one of the most burdensome and expensive diseases of the 21st century [1,2]. It is considered as a continuum, with clinical stages ranging from normal to mild cognitive impairment (MCI), to dementia, illustrating that dementia is the end result of AD pathology. Progressive memory loss and cognitive dysfunction are the typical clinical symptoms of progression along the AD continuum [3,4]. With progression of the disease, patients inevitably and gradually lose their ability to lead an independent life [5,6]. The complex interactive effects of environmental and genetic factors may affect the disease etiology and patient trajectory along the AD continuum. Advanced age (older than 65 years) and presence of the apolipoprotein E (APOE) ε4 allele have been deemed the strongest risk factors for AD [7]. However, the contribution of APOE may account for less than 20% of the risk of late-onset Alzheimer’s disease (LOAD) [8]. It is known that amyloid β (Aβ) plaques and phosphorylated-tau-containing neurofibrillary tangles are the essential pathological bases of AD [9,10], and alterations of vessels [11] and neuroinflammation [12] are considered to act in parallel to, or upstream of, Aβ accumulation.

There have, recently, been great advancements in the identification of biomarkers, and the recognition of multiple protective and causative genes. Neuroimaging biomarkers play important roles in AD. Medial temporal lobe (especially hippocampus) atrophy on MRI, Aβ deposition on amyloid-PET, and posterior cingulate (PCC) hypometabolism on ^18^fluorodeoxyglucose (^18^FDG)-PET have been validated for the diagnosis of AD [5,13]. It was revealed by voxel-based morphometry (VBM) that the brain atrophy in AD patients begins in the entorhinal cortex, and then gradually involves the hippocampus [14]. This progression of atrophy is consistent with the typical pattern of increased neurofibrillary tangles, described by Braak [15]. Changes in functional connectivity between the hippocampus and PCC have been observed prior to CSF Aβ42 abnormality [16]. Grothe et al. [17] found that Aβ deposition began in the temporobasal and frontomedial areas, and then gradually affected the neocortex, primary sensorimotor areas, medial temporal lobe, and finally the striatum. Representative neural circuits in the AD continuum have been found, including bilateral caudate–rostral middle frontal gyrus (rMFG), putamen–rMFG, and hippocampus–PCC [18,19,20]. Meina Quan et al. [21] found that amyloid precursor protein (APP) and presenilin 1 (PS1) gene mutations affected frontostriatal circuits in familial AD, and general cognitive function was associated with the structural connectivity of both frontostriatal, and hippocampus–PCC circuits.

Large genome-wide association studies (GWASs) have discovered more than 40 alleles associated with risk of AD [5,22]. The phosphatidylinositol-binding clathrin assembly protein (PICALM) gene is considered one of the most significant susceptibility factors for LOAD, ranking third after APOE, and Bridging integrator 1 gene (BINI) [23,24]. Immunological studies have found that the PICALM protein predominately exists in brain capillary endothelial cells [25,26]. It plays a crucial role in clathrin-mediated endocytosis. Previous studies using animal and cell models have reported that PICLAM modulates tau degradation [27,28], and regulates amyloid precursor protein (APP) and amyloid β transcytosis [29,30]. In a murine model, Zhen et al. [30] found that PICALM deficiency decreased the Aβ clearance across the blood–brain barrier (BBB), and accelerated Aβ pathology. They also proved that Aβ clearance and PICALM levels were reduced in AD-derived endothelial monolayers. In addition, PICALM influenced the ratio of Aβ42 to total Aβ in neurons via clathrin-mediated endocytosis of γ-secretase [29]. A variant of PICALM, rs3851179, a single-nucleotide polymorphism (SNP) at the 5′ end of the PICALM gene, is significantly associated with risk of AD. Compared with the rs3851179 G-allele, the A-allele is associated with a lower risk of AD [23,31]. Furthermore, the rs3851179 A-allele is considered a protective allele, associated with a higher Aβ clearance ratio, and higher expression levels of PICALM mRNA [30]. However, there is a lack of definitive clinical evidence regarding the differences in Aβ and tau levels between living humans with different PICALM rs3851179 genotypes.

Previous studies have indicated that this risk-associated gene may influence brain structure and function. Yang et al. [32] found that the interactive effect between PICALM rs3851179 and CLU rs11136000 influenced hippocampal volume, and hippocampal shape. Katrin et al. [33] proved that PICALM rs3851179 genotypes modulate the brain atrophy in APOE ε4 carriers. Peng Zhang et al. [34] indicated that PICALM G-carriers showed weaker negative functional connectivity of the hippocampus than A-carriers in a group of young adults, but there was no difference in the gray matter volume between the two genotype groups. However, previous studies have either focused on one stage of AD continuum or merely recruited young healthy people, and thus have overlooked the progression of AD, and may have obscured the genetic influence on disease progression. 

It is not clear yet whether different PICALM rs3851179 genotypes affect AD progression, and there is an urgent need to explore the underlying biological mechanism connecting the AD continuum, and PICALM rs3851179 genotypes. The purpose of the present study was to analyze whether the genotypes of PICALM rs3851179 were related to AD brain atrophy, Aβ level, and tau level of CSF. In this study, we focused on the main effect of PICALM, and the interactive effect between PICALM and AD continuum stage on cortex thickness, amyloid β level, and tau level. 

## 2. Materials and Methods 

### 2.1. Participants 

This study recruited cognitively normal (CN) controls, MCI patients, and early LOAD patients from the Alzheimer’s Disease Neuroimaging Initiative (ADNI) project (http://adni.loni.usc.edu, accessed on 1 May 2022). All subjects were derived from ADNI1 baseline data. The baseline structure magnetic resonance images (sMRI) and clinical data of ADNI 1 dataset were downloaded from the Image Data Archive at Laboratory of Neuro Imaging (IDA, https://ida.loni.usc.edu/login.jsp, accessed on 1 May 2022). Consent was obtained from all subjects, according to the declaration of Helsinki. IEC (Independent Ethics Committee) for Clinical Research of the First Affiliated Hospital of Soochow University approved the protocol, and the approval number was 2019 the 71st.

The subjects were recruited according to the following criteria: (1) with T1-weighted structural MRI (sMRI); (2) Age ≥ 65 years; (3) with APOE status (ε4 allele carriers/ε4 allele non-carriers), and PICALM (rs3851179) genotypes (AA/AG/GG) recorded by genetic sequencing. 

The CN, MCI, and AD subjects were diagnosed according to the diagnostic report in the ADNI database [35]. CN controls: (1) without memory complaints; (2) the Clinical Dementia Rating (CDR) = 0; (3) the Mini-Mental State Exam score (MMSE) between 24 and 30 (inclusive); (4) cognitively normal, based on an absence of significant impairment in cognitive functions or activities of daily living. MCI: (1) with memory complaints; (2) CDR = 0.5; (3) MMSE between 24 and 30 (inclusive); (4) general cognition and functional performance sufficiently preserved. AD: (1) with memory complaints; (2) CDR was 0.5 with a mandatory requirement of memory box score being 0.5 or greater; (3) MMSE between 20 and 26 (inclusive); (4) Meeting the National Institute of Neurological and Communicative Disorders and Stroke–Alzheimer’s Disease and Related Disorders Association (NINCDS/ADRDA) criteria for probable AD. 

The exclusion criteria were as follows: with significant neurologic disease; neuroimaging found evidence of infection, infarction or other focal lesions; with psychiatric disorders; with alcohol abuse; with significant medical illness; sMRI data with obvious motion or susceptibility artifacts, or with lower cortical segment processing quality. 

A total of 188 CN controls, 261 patients with MCI, and 140 patients with LOAD were finally enrolled in the study. In CN group, there were 25 individuals carrying the AA genotype of the PICALM rs3851179, 92 individuals carrying the AG genotype, and 71 individuals carrying the GG genotype. In the MCI group, there were 28 individuals carrying the AA genotype of the PICALM rs3851179, 119 individuals carrying the AG genotype, and 114 individuals carrying the GG genotype. In AD patients, 17 subjects carried the AA genotype of PICALM rs3851179, 54 subjects carried the AG, and 69 subjects carried the GG. Each group was, respectively, divided into an A-carrier subgroup (including AA homozygote and AG heterozygote) and a GG-carrier subgroup [33].

All the subjects underwent neuropsychological tests. The dementia severity was evaluated via the Alzheimer’s Disease Assessment Scale Cognitive-13 (ADAS13); the cognitive impairment was evaluated via MMSE; the memory function was evaluated via the Rey Auditory Verbal Learning Test immediate recall (RAVLT immediate). A total of 290 participants (93 in CN, 128 in MCI, and 69 in AD) accepted lumbar puncture. The cerebrospinal fluid (CSF) levels of Aβ42, Aβ40, Aβ38, total tau (T-tau), and phosphorylated tau (P-tau) were analyzed in this study. The ratio of Aβ42 to Aβ40 (Aβ42/40), and the ratio of P-tau to T-tau (P/T-tau) were also calculated and analyzed.

### 2.2. MRI Data Acquisition and Processing 

The baseline sMRI data were obtained from the ADNI 1 database on 1.5T scanner using three-dimensional magnetization prepared rapid acquisition gradient-echo (MPRAGE) image sequences. More details of the sMRI were available at the website (https://adni.loni.usc.edu/methods/documents/, accessed on 1 May 2022). 

The surface-based morphometry (SBM) of sMRI data was processed on Computational Anatomy Toolbox (CAT12, version: r1742), and Statistical Parametric Mapping (SPM12: https://www.fil.ion.ucl.ac.uk/spm/software/spm12/, accessed on 1 May 2022). The sMRI images of all subjects were segmented and normalized into Montreal Neurological Institute (MNI) space using Diffeomorphic Anatomical Registration Through Exponentiated Lie Algebra (DARTEL algorithm) [36]. Those images were segmented into gray matter, white matter, and CSF. After affine registration of MNI space and non-linear deformation, the data of cortex thickness were smoothed by a 12 mm Full Width at Half Maximum (FWHM).

Before data analysis, cortical segment processing was estimated and graded by CAT12 quality reports, which included image homogeneity covariance test, resolution, noise, and bias estimation. Subjects with a grade lower than “C+” were excluded. 

### 2.3. Statistical Analyses

#### 2.3.1. MRI Data Analysis

There were two factors in the study. The CN, MCI, and AD patients, respectively, represent three different stages in the AD continuum, which were defined as Disease factor. PICALM genotypes (rs3851179 A-carriers and GG-carriers) were defined as PICALM factor.

The effects of the two factors on cortex thickness were evaluated in SPM12 using a full factorial analysis of covariance (ANCOVA) with age, sex, education years, and APOE ε4 status as covariates. Multiple comparisons were corrected by Family Wise Error (FWE) at cluster level (correction threshold *p* < 0.05, peak-level threshold *p* < 0.001). The following results were obtained from the statistical model: (1) the interactive effect between PICALM and Disease (PICALM × Disease); (2) the main effect of PICALM; (3) the main effect of Disease. If the interactive effect between PICALM × Disease was significant, the simple effect was further analyzed. If not, the main effects of PICALM and Disease were further analyzed.

To exclude the interactive effect of PICLAM genotypes and APOE ε4 status, a full factorial ANCOVA was designed as a supplementary trial to assess the effects of the three factors (PICALM × Disease × APOE) on cortex thickness, with age, sex, and education years as covariates. Multiple comparisons were corrected by FWE at cluster level.

The “significant clusters” were defined as the brain areas with significant differences after FWE correction. The significant clusters were reported by Desikan–Killiany (DK40) atlas, and the Human Connectome Project Multi-Modal Parcellation (HCP MMP) atlas [37,38] was further used to reflect the functional regions accurately, if necessary. The “peak vertex” was with the greatest statistical differences in the significant cluster, and the MNI coordinates of peak vertex were reported.

#### 2.3.2. Clinical Data and Demographic Characteristics 

The clinical data and demographic characteristics were analyzed by the Statistical Program for Social Science (SPSS, version 21.0). For clinical data, the effects of PICALM factor and Disease factor on MMSE, ADAS13, RAVLT immediate, Amyloid β (including Aβ42, Aβ40, Aβ38 and Aβ42/40), and tau (including T-tau, P-tau and P/T-tau) were also assessed by a full factorial ANCOVA, using the Least Significance Difference (LSD) post hoc test and with age, sex, education years, and APOE ε4 status as covariates.

For demographic characteristics, the two samples t-test was used to analyze the differences in education years and age, between PICALM subgroups of CN, MCI, and LOAD groups, respectively. The Chi-square test was used to analyze the differences in gender, and APOE ε4 status. A *p*-value less than 0.05 was considered significant.

#### 2.3.3. Correlation Analysis

The mean cortex thickness values were extracted from each significant cluster by CTA12. The correlations between cortex thickness and CSF biomarkers were analyzed via Spearman’s correlation in SPSS. A *p*-value less than 0.05 was considered significant. 

## 3. Results

### 3.1. MRI Data Findings

#### 3.1.1. The Interactive Effect of PICALM and Disease on Cortex

The PICALM × Disease interactive effect on cortex thickness was not significant, indicating the PICALM factor and Disease factor were independent from each other.

#### 3.1.2. The Main Effects of PICALM on Cortex

**Figure 1:**
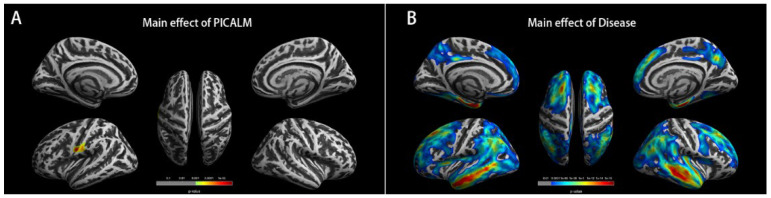
The main effects analysis. Note: (**A**) The brain map represents the main effect of PICALM factor on cortex thickness. The cortex thickness of left postcentral cortex is significantly different between rs3851179 A-carriers and GG-carriers after FWE correction (cluster level). (**B**) The brain map represents the main effect of Disease factor on cortex thickness. The cortex thicknesses of bilateral temporal and prefrontal cortex are the most significantly different among CN, MCI, and LOAD group.

**Figure 2:**
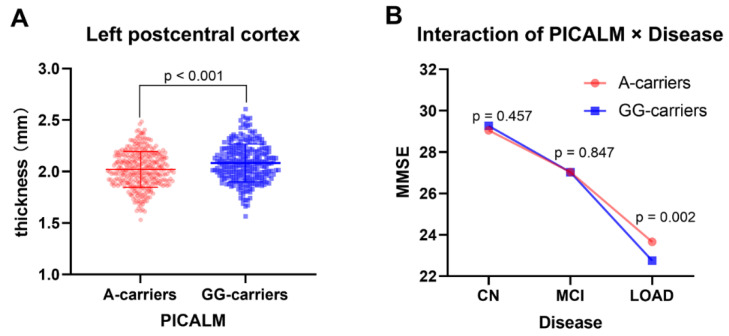
The main effect of PICALM on left postcentral cortex and the interaction of PICALM and Disease on MMSE. Note: (**A**), the thickness of left postcentral cortex was extracted from A-carriers and GG-carriers. The mean value of GG-carriers is higher than that of A-carriers. (**B**), the interactive effect of PICALM and Disease on MMSE is significant (F = 4.539, *p* = 0.011). In LOAD patients, a higher MMSE score is found in A-carriers, compared with GG-carriers (*p* = 0.002). While in CN or MCI patients, MMSE is not significant different between A-carriers and GG-carriers (*p* = 0.457, 0.847, respectively). The MMSE score decreases significantly in both A-carriers and GG-carriers, from CN to MCI to LOAD.

The main effects of PICALM were further analyzed for the subjects (including CN, MCI, and LOAD individuals). The main effects of PICALM on cortex thickness were significantly different (F = 21.0, *p* < 0.001). As shown in Figure 1A and Table 1, the peak vertex was located on the left postcentral cortex (in DK40 atlas), and the significant clusters were expanded to the left precentral cortex. In the HCP MMP atlas, the peak vertex was located in left area 1, and the significant cluster were expanded to left area 3a, area OP4, area 3b, area 4, area PFop, and area 2. The thickness of left postcentral cortex was extracted from A-carriers and GG-carriers, and the mean value of GG-carriers (2.082 ± 0.183 mm) was significantly higher than that of A-carriers (2.021 ± 0.174 mm), as shown in Figure 2A.

#### 3.1.3. The Main Effect of Disease on Cortex

As shown in Figure 1B and Supplementary Table S1, the main effects of Disease on cortex were significant and broad. The cortex thicknesses of bilateral, temporal, and prefrontal cortex were most significantly different among CN, MCI, and LOAD groups. There were two significant clusters in the left hemisphere. The peak vertex of the larger one was located in the left middle temporal, entorhinal, and superior parietal (MTP-entorhinal-SP) cortex, and the significant clusters were expanded to the left fusiform, posterior cingulate, frontal, parietal, and temporal lobes. The peak vertex of the smaller one was located in the left lateral orbitofrontal cortex. There were three significant clusters on the right hemisphere. The peak vertex is, respectively, located in the right middle temporal and entorhinal (MTP-entorhinal) cortex, right superior frontal cortex and right fusiform cortex, and the significant clusters were expanded to the right frontal, parietal, and temporal lobes. 

#### 3.1.4. The Interactive Effect of PICALM × Disease × AOPE on Cortex

The supplementary full factorial ANCOVA demonstrated that there were no significant interactive effects of PICALM × Disease × AOPE, PICALM × AOPE or Disease × AOPE. The main effect of APOE status was not significant either. 

### 3.2. Demographic and Clinical Data Findings

#### 3.2.1. The Results of Demographic Data

As shown in Table 2, there were no significant differences in APOE ε4 status, gender, age, and education years between A-carriers and GG-carriers of CN, MCI, and LOAD groups, respectively (*p* > 0.05).

#### 3.2.2. The Interactive Effect of PICALM × Disease on Clinical Data

The interactive effect of PICALM × Disease on MMSE was significant (F = 4.539, *p* = 0.011). As shown in Figure 2B, in the A-carriers subgroup, the MMSE significantly decreased from CN to MCI to AD (*p* < 0.001). In the GG-carriers subgroup, the MMSE also significantly decreased from CN to MCI to AD (*p* < 0.001). In AD patients, MMSE scores were higher in A-carriers than in GG-carriers (*p* = 0.002), whereas in CN and MCI patients, MMSE scores were not significantly different between A-carriers and GG-carriers (*p* > 0.05). There were no significant interactive effects of PICALM × Disease on Aβ42, Aβ40, Aβ38, Aβ42/40, T-tau, P-tau, P/T-tau, ADAS13, and RAVLT immediate (*p* > 0.05).

#### 3.2.3. The Main Effect of Disease on Clinical Data

As shown in Table 3, the neuropsychological tests (ADAS13 and RAVLT immediate) deteriorated significantly from CN to MCI to LOAD (*p* < 0.001). The CN subjects had higher Aβ42 level and Aβ42/40 ratio, and lower T-tau level, P-tau level, and P/T-tau ratio than MCI and LOAD patients (*p* < 0.05). The differences in Aβ40 and Aβ38 levels among CN, MCI, and LOAD were not significant (*p* > 0.05). The differences in Aβ42 level, Aβ42/40 ratio, T-tau level, P-tau level, and P/T-tau ratio between MCI and LOAD were also not statistically significant (*p* > 0.05). 

#### 3.2.4. The Main Effect of PICALM on Clinical Data

As shown in Table 4, the A-carriers had higher Aβ42 level and Aβ42/40 ratio than GG-carriers, and lower P/T-tau ratio than GG-carriers (*p* < 0.05). There were no statistical differences in ADAS13, RAVLT immediate, Aβ40, Aβ38, T-tau, and P-tau levels between A-carriers and GG-carriers (*p* > 0.05).

### 3.3. The Results of Correlation Analysis

As shown in Supplementary Table S2, the left MTP-entorhinal-SP, right MTP-entorhinal, right fusiform, and right superior frontal cortexes were significantly positively correlated with Aβ42 level (*p* < 0.05, *r* = 0.167, 0.172, 0.161 and 0.156, respectively) and Aβ42/40 ratio (*p* < 0.05, *r* = 0.186, 0.177, 0.135 and 0.170, respectively), while also being negatively correlated with P-tau level (*p* < 0.05, *r* = −0.271, −0.285, −0.201 and −0.224, respectively) and T-tau level (*p* < 0.05, *r* = −0.283, −0.295, −0.211 and −0.238, respectively). The left MTP-entorhinal-SP and right MTP-entorhinal cortex thickness also correlated with P/T-tau ratio (*p* < 0.05, *r* = −0.117 and −0.131, respectively), while the right fusiform and right superior frontal cortex thicknesses were not correlated with P/T-tau ratio (*p* > 0.05). The right preoperculars thickness positively correlated with Aβ42 level (*p* = 0.022, *r* = 0.134), and didn’t correlate with Aβ42/40 ratio, P-tau level, T-tau level or P/T-tau ratio (*p* > 0.05). The right posterior cingulate thickness was positively correlated with Aβ42 level (*p* = 0.022, *r* = 0.134) and negatively correlated with P-tau level (*p* = 0.028, *r* = −0.130) and T-tau level (*p* = 0.020, *r* = −0.138), while it was not correlated with Aβ42/40 ratio or P/T-tau ratio (*p* > 0.05). The left postcentral thickness and left lateral orbitofrontal thickness were not correlated with Aβ42 level, Aβ42/40 ratio, P-tau level, T-tau level or P/T-tau ratio (*p* > 0.05).

## 4. Discussion 

The PICALM gene was considered one of the genes most linked to LOAD susceptibility [23,24]. Previous studies, using animal and cell models, reported that PICLAM modulated tau [27,28] and Aβ [29,30]. However, such reports are lacking in living humans. The effect of different PICALM genotypes on cortical atrophy in AD progression remains unclear. 

The previous studies emphasized the interactions of risk genes [32,33,34], while the present study focused on the PICALM genotypes, and AD continuum stages. Previous studies have indicated that PICLAM and APOE affected the gray matter volume in patients with AD, and the homozygous G-allele and APOE ε4 exerted an adverse effect on prefrontal volume [33]. However, in our supplementary test, there was no significant interaction between PICALM and APOE on cortex thickness, which was not consistent with the previous study, and may be caused by the heterogeneity of the subjects. The subjects in our study were over 65 years old. Additionally, the difference in SBM and VBM methods may also affect the results. The main effects of AD continuum on cortex were broad, which was consistent with previous study. Bilateral temporal lobe atrophy was the most significant [5].

It was an interesting finding that PICALM rs3851179 may affect cortex thickness. GG-carriers have a thicker left postcentral cortex than A-carriers, which has not been reported in previous neuroimaging studies. In the 1930s, Penfield et al. [39] used electrical stimulation to map somatosensory cortex areas, and the postcentral gyrus was considered to play a central role in processing sensory information from the body. The somatosensory cortex is divided into the primary and second somatosensory cortex, and the primary somatosensory area is located in the postcentral cortex [40]. The postcentral cortex corresponds to Brodmann areas 3, 2 and 1, with area 3 being further divided into 3a and 3b. The second somatosensory corresponds to Brodmann areas 40 and 43, and the OP4 is contained in Brodmann area 43 [41]. The brain areas of HCP MMP were similar to those of Brodmann atlas, but the former is more suitable for describing cortex regions. In our study, the significant cluster of PICALM was mainly constituted by HCP MMP areas 1, 3a, 3b and OP4, which included the primary and secondary somatosensory areas. Recent studies have shown that the somatosensory cortex also affects various stages of emotional processing [42], such as stimulating emotional identification [43], generating emotional states [44], and regulating emotions [45]. 

In LOAD patients, rs3851179 A-carriers had higher MMSE scores than GG-carriers. However, in CN and MCI patients, the difference was not significant. It was indicated that the rs3851179 A-allele may be protective for cognitive function in the dementia stage. Neuropsychological condition gradually deteriorated from CN to MCI to LOAD in the study, which was consistent with previous studies.

The Aβ42, T-tau, and P-tau are core biomarkers of AD, especially Aβ42 [46,47]. In this study, the CSF Aβ42 concentration decreased significantly in MCI and AD patients than in the CN group, which was consistent with previous studies [48,49,50]. Lower CSF Aβ42 levels reflect the deposition of amyloid plaques in the brain. In other words, higher CSF Aβ42 levels imply lower amyloid plaques. [51]. Previous studies in mice have also demonstrated that PICLAM may regulate the Aβ42 level [29,30]. Zhao et al. [30] found the rs3851179 protective A-allele exhibited higher Aβ clearance and PICALM mRNA level in inducible pluripotent stem cell-derived human endothelial cells. Encouragingly, in the study, the theory was tested in living humans. CSF Aβ42 levels and Aβ42/40 ratio were higher in A-carriers than in GG-carriers, indicating that the A-allele can reduce Aβ42 deposition in the brain, which supports that the A-allele is a protective gene for AD. The Aβ40 is the second most studied amyloid protein after Aβ42. Previous meta-analysis found a small difference in Aβ40 levels between AD and CN, no difference in Aβ40 level between MCI and CN, and no difference in Aβ38 level among CN, MCI, and AD [50]. In our study, the differences in Aβ40 level and Aβ38 level were not significant among CN, MCI, and LOAD.

Previous studies found that CSF T-tau and P-tau levels are elevated, and Aβ42 level is decreased in MCI and AD patients [50,52]. In the present study, CSF T-tau and P-tau levels, and P/T ratio increased significantly in MCI and LOAD patients. PICLAM protein regulates tau degradation [27,28]. Kunie et al. [27] demonstrated that PICLAM low expression aggravated tau protein pathologies, and tau-mediated neurodegeneration in vivo mice. Zhao’s research [30] found the rs3851179 A-carrying mice have higher PICALM mRNA level than G-carrying mice. In our study, the P/T-tau ratio was significantly lower in rs3851179 A-carriers than in GG-carriers, which supported a protective effect of the PICALM rs3851179 A-allele. A-allele carriers had lower P-tau and T-tau than GG-carriers, but the differences were not significant. There was no significant interactive effect between PICALM and Disease, indicating that the effects of PICLAM rs3851179 on Aβ and tau were not related to AD continuum stages.

In this study, PICALM rs3851179 genotypes may modulate the left postcentral cortex, and CSF Aβ42 level and P/T-tau ratio. We further analyzed the correlation between the left postcentral cortex and CSF Aβ42 level, as well as the correlation between the left postcentral cortex and tau levels. Unfortunately, in the left postcentral cortex, the main effect of PICLAM was not correlated with Aβ42 or tau levels, whereas most cortexes of AD continuum main effect were correlated with Aβ42 or tau levels. It was indicated that PICLAM may not affect cortex thickness via Aβ and tau pathways. Recently, the potential pathway of PICALM in AD can be roughly divided into Aβ-dependent, and Aβ-independent pathways. In addition to tauopathy, the latter pathway included synaptic dysfunction [53], disorganized lipid metabolism [30], immune disorder [54], and disrupted iron homeostasis [55]. In short, the specific mechanism of PICALM remains unclear. However, some researchers have demonstrated that PICLAM can influence the brain structure, function, and biomarkers. Our study also suggested a potential effect of PICALM on human brain structure. The left lateral orbitofrontal thickness was one brain area showing the main Disease effect in this study, but also it did not correlate with Aβ and tau. It suggested a potential pathway of brain atrophy without Aβ and tau.

There were some limitations in this study. First, it was not precise that we used CN, MCI, and AD subjects to represent the three stages of the AD continuum. A better method would be to design a longitudinal study with a larger sample and long-term follow-up to observe how genes affect the AD progression, from normal cognition to MCI to dementia. However, this would consume a lot of resources. We also didn’t find enough samples with long-term follow-up and gene sequencing in ADNI. Second, the AA homozygotes are a small sample of the population. The rs3851179 A-carriers in our study comprised AA homozygotes, and AG heterozygotes. Although a previous study set up similar subgroups [33], the potential influence of the G allele in AG heterozygotes needs to be further investigated. Third, the main effect of PICALM was on the left postcentral cortex, which played a crucial role in processing sensory information, and regulating emotion. However, there were no somatosensory and emotional tests in this study. It is necessary to explore the relationship between rs3851179 genotypes, and somatosensory and emotional function in the future.

## 5. Conclusions

In conclusion, the interactive effects of PICALM and the AD continuum on cortex and CSF biomarkers were not significant, suggesting that the main effects of PICALM were not related to AD continuum stages. The cortex thickness and CSF biomarkers were significantly different between A-carriers and GG-carriers, indicating that PICLAM rs3851179 genotypes may modulate the left postcentral cortex thickness, Aβ42 level, and P/T-tau ratio. The rs3851179 A-allele may have a protective effect, reflected by increased CSF Aβ42 level, and reduced P/T-tau ratio in the A-carriers. The interactive effects of PICALM and AD continuum on MMSE score demonstrated that the rs3851179 A-allele may protect the cognitive function of LOAD patients.

## Figures and Tables

**Table 1:** The main effects of PICALM on cortex thickness.

Cluster Peak *p*-Values	Cluster Peak F-Score	Cluster Size	MNI Coordinates			Overlap of Atlas Region	
			x	y	z	DK40 Atlas	HCP MMP Atlas
0.00001	21.0	1336	−59	−5	17	postcentral L	area_1 L
						precentral L	area_3a L
							OP4 L
							area_3b L
							PFop L
							area_2 L

Note: The peak *p*-value, peak F-score and MNI (Montreal Neurological Institute) coordinates (x, y, z) of the peak vertex are reported. The DK40 atlas and HCP MMP atlas are used to report the brain regions with significantly different cortex thickness. The postfix “L” means the cluster located in left hemisphere.

**Table 2:** The differences in demographic data between A-carriers and GG-carriers of CN, MCI, and LOAD groups.

	CN			MCI			LOAD		
	A-Carriers (n = 117)	GG-Carriers (n = 71)	*p* Value (*x*^2^/T Value)	A-Carriers (n = 147)	GG-Carriers (n = 114)	*p* Value (*x*^2^/T Value)	A-Carriers (n = 71)	GG-Carriers (n = 69)	*p* Value (*x*^2^/T value)
APOE ε4 (carriers/noncarriers)	84/33	51/20	0.996 (<0.001)	72/75	45/69	0.126 (2.346)	24/47	18/51	0.319 (0.992)
Gender (males/females)	62/55	34/37	0.497 (0.461)	94/53	74/40	0.872 (0.026)	38/33	35/34	0.741 (0.110)
Age (years)	75.92 ± 4.57	75.89 ± 4.73	0.932 (−0.086)	76.72 ± 5.25	75.56 ± 5.90	0.096 (1.672)	77.47 ± 6.04	76.73 ± 5.62	0.457 (0.745)
Education (years)	15.73 ± 2.73	16.15 ± 2.93	0.311 (−1.015)	15.60 ± 2.93	15.81 ± 3.35	0.593 (−0.535)	14.69 ± 3.05	14.22 ± 3.42	0.389 (0.864)

Note: APOE, apolipoprotein E; CN, cognitive normal; MCI, mild cognitive impairment; LOAD, late-onset Alzheimer’s disease.

**Table 3:** Main effects of Diseases on neuropsychological tests, amyloid β, and tau.

Characteristic	CN	MCI	LOAD	Statistics	*p* Value	CN vs. MCI *p* Value	CN vs. LOAD *p* Value	MCI vs. LOAD *p* Value
ADAS13	9.59 ± 4.39	18.74 ± 5.93	28.96 ± 6.82	F = 357.789	<0.001	<0.001	<0.001	<0.001
RAVLT immediate	42.98 ± 9.3	30.66 ± 8.93	23.12 ± 7.47	F = 169.504	<0.001	<0.001	<0.001	<0.001
Amyloid β42 (pg/mL)	1156.43 ± 555.11	797.68 ± 426.07	691.86 ± 343.97	F = 9.797	<0.001	<0.001	<0.001	0.629
Amyloid β40 (pg/mL)	7777.81 ± 2590.03	7746.04 ± 2059.81	7446.2 ± 2418.3	F = 0.798	0.451			
Amyloid β42/40	0.151 ± 0.054	0.105 ± 0.051	0.094 ± 0.034	F = 14.621	<0.001	<0.001	<0.001	0.814
Amyloid β38 (pg/mL)	1843.13 ± 630.32	1811.48 ± 525.88	1721.26 ± 595.69	F = 0.907	0.405			
T-tau (pg/mL)	236.87 ± 86.63	313.74 ± 122.17	352.35 ± 122.43	F = 12.410	<0.001	<0.001	<0.001	0.207
P-tau (pg/mL)	22.06 ± 9.17	31.09 ± 14.12	35.21 ± 13.89	F = 12.328	<0.001	<0.001	<0.001	0.248
P/T-tau	0.092 ± 0.007	0.097 ± 0.009	0.099 ± 0.009	F = 4.141	0.017	0.010	0.015	0.754

Note: ADAS13, Alzheimer’s Disease Assessment Scale Cognitive-13; RAVLT-immediate, Rey Auditory Verbal Learning Test immediate recall; P-tau, phosphorylated tau; T-tau, total tau; P/T-tau, the ratio of phosphorylated tau to total tau. CN, cognitive normal; MCI, mild cognitive impairment; LOAD, late-onset Alzheimer’s disease.

**Table 4:** Main effects of PICALM on neuropsychological tests, amyloid β, and tau.

Characteristic	A-Carriers	GG-Carriers	Statistics	*p* Values
ADAS13	17.63 ± 8.68	18.9 ± 9.70	F = 0.026	0.871
RAVLT-immediate	33.1 ± 11.16	32.41 ± 12.04	F = 0.440	0.508
Amyloid β42 (pg/mL)	954.7 ± 531.58	807.17 ± 427.31	F = 4.335	0.038
Amyloid β40 (pg/mL)	7678.27 ± 2322.57	7692.8 ± 2331.27	F = 0.012	0.914
Amyloid β42/40	0.125 ± 0.055	0.108 ± 0.050	F = 4.733	0.039
Amyloid β38 (pg/mL)	1804.39 ± 588.23	1795.11 ± 566.83	F = 0.020	0.889
T-tau (pg/mL)	283.37 ± 109.25	316.03 ± 130.67	F = 2.110	0.147
P-tau (pg/mL)	27.25 ± 12.14	31.45 ± 14.98	F = 3.164	0.076
P/T-tau	0.094 ± 0.009	0.097 ± 0.009	F = 4.765	0.030

Note: APOE, apolipoprotein E; ADAS13, Alzheimer’s Disease Assessment Scale Cognitive-13; RAVLT-immediate, Rey Auditory Verbal Learning Test immediate recall; P-tau, phosphorylated tau; T-tau, total tau; P/T-tau, the ratio of phosphorylated tau to total tau.

## Data Availability

All data in this study are available in Alzheimer’s Disease Neuroimaging Initiative (ADNI) project (http://adni.loni.usc.edu, accessed on 1 May 2022).

## Supplementary Materials

The following supporting information can be downloaded at: https://www.mdpi.com/article/10.3390/brainsci12121681/s1. Table S1 The main effect s of Disease on cortex thickness. Table S2 The correlation s between significant cluster s and CSF biomarkers.
