# Supplementary material for: PICALM rs3851179 Variants Modulate Left Postcentral Cortex Thickness, CSF Amyloid β42, and Phosphorylated Tau in the Elderly
Source: Brain Sci. 2022 Dec 7;12(12):1681. doi: 10.3390/brainsci12121681 (PMC9776362; doi:10.3390/brainsci12121681)
Supplement: Supplementary file 1 [file brainsci-12-01681-s001.zip › brainsci-2032063-supplementary.pdf]

**Supplementary Table S1. The main effects of Disease on cortex thickness.**

| Main effects on left/right hemisphere | Cluster P values | F value | Cluster size | Overlap of atlas region |
|---|---|---|---|---|
| Left hemisphere | 0.00000 | 49.8 | 79747 | 10% superiorfrontal |
| | | | | 9% inferiorparietal |
| | | | | 9% **superiorparietal** |
| | | | | 8% supramarginal |
| | | | | 8% rostralmiddlefrontal |
| | | | | 7% superiortemporal |
| | | | | 6% **middletemporal** |
| | | | | 5% lateraloccipital |
| | | | | 5% caudalmiddlefrontal |
| | | | | 4% precuneus |
| | | | | 4% inferiortemporal |
| | | | | 3% fusiform |
| | | | | 3% precentral |
| | | | | 3% insula |
| | | | | 2% bankssts |
| | | | | 2% parsopercularis |
| | | | | 2% parahippocampal |
| | | | | 2% parstriangularis |
| | | | | 1% **entorhinal** |
| | | | | 1% posteriorcingulate |
| | | | | 1% medialorbitofrontal |
| | | | | 1% lateralorbitofrontal |
| | | | | 1% temporalpole |
| | 0.00000 | 18.6 | 770 | 100% **lateralorbitofrontal** |

| | | | | |
|---|---|---|---|---|
| Right hemisphere | 0.00000 | 48.3 | 49850 | 19% inferiorparietal |
| | | | | 12% superiortemporal |
| | | | | 12% superiorparietal |
| | | | | 10% **middletemporal** |
| | | | | 10% precuneus |
| | | | | 9% supramarginal |
| | | | | 6% lateraloccipital |
| | | | | 5% inferiortemporal |
| | | | | 4% bankssts |
| | | | | 3% insula |
| | | | | 2% **entorhinal** |
| | | | | 2% fusiform |
| | | | | 2% temporalpole |
| | | | | 2% transversetemporal |
| | | | | 2% paracentral |
| | | | | 1% parahippocampal |
| | 0.00000 | 28.2 | 19631 | 41% **superiorfrontal** |
| | | | | 27% rostralmiddlefrontal |
| | | | | 15% caudalmiddlefrontal |
| | | | | 12% precentral |
| | | | | 2% medialorbitofrontal |
| | | | | 2% parsopercularis |
| | 0.00000 | 22.6 | 2214 | 77% **fusiform** |
| | | | | 17% lingual |
| | | | | 6% parahippocampal |
| | 0.00000 | 16.6 | 1519 | 41% **parsopercularis** |

| | | | | |
|---|---|---|---|---|
| | | | | 30% insula |
| | | | | 21% precentral |
| | | | | 7% parstriangularis |
| | 0.00003 | 10.7 | 513 | 86% **posteriorcingulate** |
| | | | | 13% precuneus |
| | | | | 1% isthmuscingulate |

Note: The text in bold means the brain area with peak difference in the cluster.

**Supplementary Table S2. The correlations between significant clusters and CSF biomarkers.**

| | Amyloid β42<br>p value (r value) | Amyloid β42/40<br>p value (r value) | P-tau<br>p value (r value) | T-tau<br>p value (r value) | P/T-tau<br>p value (r value) |
|---|---|---|---|---|---|
| Postcentral L | 0.512 (-0.039) | 0.979 (0.002) | 0.648 (-0.027) | 0.430 (-0.047) | 0.372 (0.053) |
| MTP-entorhinal-SP L | **0.004 (0.167)** | **0.002 (0.186)** | **< 0.001 (-0.271)** | **< 0.001 (-0.283)** | **0.048 (-0.117)** |
| lateralorbitofrontal L | 0.386 (0.051) | 0.787 (0.016) | 0.277 (-0.064) | 0.215 (-0.074) | 0.816 (-0.014) |
| MTP-entorhinal R | **0.003 (0.172)** | **0.003 (0.177)** | **< 0.001 (-0.285)** | **< 0.001 (-0.295)** | **0.026 (-0.131)** |
| fusiform R | **0.006 (0.161)** | **0.021 (0.135)** | **0.001 (-0.201)** | **< 0.001 (-0.211)** | 0.168 (-0.082) |
| superiorfrontal R | **0.008 (0.156)** | **0.004 (0.170)** | **< 0.001 (-0.224)** | **< 0.001 (-0.238)** | 0.074 (-0.106) |
| parsopercularis R | **0.022 (0.134)** | 0.628 (0.029) | 0.618 (-0.030) | 0.586 (-0.032) | 0.707 (-0.022) |
| posteriorcingulate R | **0.015 (0.143)** | 0.090 (0.100) | **0.028 (-0.130)** | **0.020 (-0.138)** | 0.270 (-0.065) |

Note: The postfix "L" means the cluster located on left hemisphere and "R" means on right hemisphere. "MTP-entorhinal-SP" represents the cluster, whose peak differences located on middletemporal, entorhinal and superiorparietal cortex. "MTP-entorhinal" cluster with peak differences on middletemporal and entorhinal cortex. P-tau: phosphorylated tau; T-tau: total tau. P/T-tau: the ratio of phosphorylated tau to total tau. The bold number means p-value was less than 0.05.
